# Fortifying alcoholic beverages with thiamine: Lessons from history and future opportunities

**DOI:** 10.1016/j.dadr.2025.100358

**Published:** 2025-07-04

**Authors:** Thomas Jedamzik, Georg Juckel

**Affiliations:** Department of Psychiatry, Ruhr University Bochum, LWL University Hospital, Alexandrinenstr. 1, Bochum D-44791, Germany

**Keywords:** Thiamine, Fortification, Korsakoff, Wernicke

## Abstract

Thiamine deficiency remains a significant risk for individuals with chronic alcohol use and is a major contributing factor in the development of Wernicke encephalopathy and Korsakoff syndrome. While clinical guidelines recommend targeted thiamine supplementation in at-risk patients, strategies for prevention at the population level remain limited and underutilized. Among the more unconventional proposals discussed in past decades was the fortification of alcoholic beverages with thiamine. This idea received particular attention in Australia in the 1980s, where high prevalence rates of Wernicke encephalopathy and Korsakoff syndrome led to a broader public health debate. Although fortification was ultimately limited to flour and cereals, the discussion raised important questions about feasibility, effectiveness, and ethical considerations, many of which remain unresolved. This commentary revisits the history of this debate, drawing on empirical studies, review articles, and opinion-based contributions published in scientific journals from the 1940s to the present. Particular attention is given to the counterarguments raised against beverage fortification, including concerns about thiamine absorption, the potential behavioral implications of such a measure, and doubts about its political and regulatory feasibility. These arguments are examined in their historical context, including how they evolved over time, what types of evidence they were based on, and how they were discussed across clinical disciplines and scientific forums. By tracing the development of this largely overlooked policy proposal, this article aims to clarify the central points of contention and encourage a more nuanced understanding of the rationale, limitations, and potential of thiamine fortification in the context of alcohol-related health risks.

## 1 Introduction

Thiamine (vitamin B1) is a water-soluble vitamin integral to cellular energy production, mitochondrial function, and neurotransmitter synthesis, serving as a coenzyme in key metabolic processes (Mrowicka et al., 2023). While beriberi, a disease caused by thiamine deficiency that affects the nervous and cardiovascular systems, has been recognized in Asia for centuries, its connection to thiamine was only established in the 1930s (Lanska, 2010). Shortly after, the chemical structure of thiamine along with a synthesis route was first published by Williams and Cline in 1936 (Williams and Cline, 1936).

In the 19th century—well before the discovery of thiamine—two distinct neuropsychiatric conditions were first described that would later be linked to its deficiency. Carl Wernicke described a syndrome characterized by the triad of an altered mental state, oculomotor abnormalities, and cerebellar dysfunction, today known as Wernicke encephalopathy (WE) (Thomson et al., 2008). Typical lesions associated with WE—atrophy, discoloration, and petechial hemorrhages of the mamillary bodies—have been identified in 0.4–2.8 % of the population (Harper et al., 1995). WE has a mortality rate of up to 17 % (Centerwall and Criqui, 1978) and is frequently overlooked, with up to 68 % of cases associated with AUD going undiagnosed and up to 94 % of cases from other origins being missed (Yoon et al., 2019).

Six years after the first description by Wernicke, Sergei Korsakoff described a syndrome defined by a combination of neurological and psychiatric symptoms now referred to as Korsakoff syndrome (KS) (Victor and Yakovlev, 1955). It is now recognized as a predominantly irreversible disorder, marked by profound episodic memory deficits while other cognitive abilities remain relatively intact. Key traits include apathy, limited insight, and, in its early phase, confabulations (Arts et al., 2017). Patients suffering from KS often require lifelong care or institutionalization, resulting in substantial costs for healthcare systems (Centerwall and Criqui, 1978).

Initially attributed to alcohol toxicity, by the 1930s it was understood that thiamine deficiency —often caused by alcohol misuse—was the common factor in both disorders. While these conditions are often collectively referred to as Wernicke-Korsakoff syndrome (WKS), their interrelation is complex and not entirely straightforward. WKS onset may be precipitated by recurring episodes of subclinical thiamine deficiency, with potential exacerbations during alcohol withdrawal (Pruckner et al., 2019).

Treatment with high doses of parenteral thiamine is recommended in acute cases of both conditions. Additionally, oral or alternative thiamine supplementation is advised for patients undergoing alcohol withdrawal (Pruckner et al., 2019). The German S3-guidelines on AUD considers the recommendation of thiamine supplementation in patients undergoing withdrawal as a point of clinical consensus. The authors of the guidelines assess the evidence on thiamine regarding its effectiveness for the prevention and treatment of WE as weak or insufficient, particularly concerning the dosage, frequency of administration, and form of delivery (DGPPN, 2020).

AUD affects thiamine levels in multiple ways: Thiamine absorption is impaired both by alcohol concentration in the intestines (Thomson et al., 1970) and by elevated blood alcohol levels (Hoyumpa, 1980). Malnutrition, common in severe AUD, further reduces thiamine absorption and adds to the effects of alcohol (Thomson, 2000). Other aspects of AUD further influence thiamine deficiency. Alcohol, being an efficient source of calories, results in a reduction of appetite and a less-varied diet. In extreme cases, alcoholic beverages become the main or sole source of caloric intake. Vomiting, diarrhea, and steatorrhea are common in AUD, furthering malnutrition and malabsorption. Furthermore, liver damage reduces the hepatic storage of thiamine. Progressive liver damage induces metabolic changes, including heightened protein demand and increased lipid utilization, that amplify metabolic strain and elevate the requirement for thiamine and other nutrients (Thomson, 2000). In addition, alcoholic beverages are low in thiamine due to fermentation and filtering processes and require a significant amount of thiamine for metabolism because of their high carbohydrate content (Price, 1985). Beyond loss and reduced resorption, metabolization of thiamine is hindered in the presence of and a result of the damage caused by alcohol (Coulbault et al., 2021).

Thiamine fortification of staple foods has been implemented in various countries (e.g., the UK, US, Canada, and Denmark), where it may have contributed to a reduced prevalence of thiamine deficiency and WKS (Harper et al., 1995). In Australia, where WKS prevalence is particularly high, the debate extended further to the possibility of fortifying alcoholic beverages directly—driven by the recognition that individuals with AUD, who are at greatest risk, may not sufficiently benefit from general food-based fortification strategies. Despite considerable discussion, however, the proposal was ultimately not implemented (Price, 1985, Wodak et al., 1990).

This commentary first revisits the historical and scientific debate on the fortification of alcoholic beverages with thiamine. It then focuses on critically examining the main objections raised against this approach—tracing how these arguments have evolved over time, clarifying misconceptions, and considering whether fortification deserves renewed attention as a complement to established supplementation efforts.

The analysis draws on a narrative synthesis of the literature retrieved via PubMed and citation tracking. Priority was given to sources explicitly addressing thiamine fortification in alcoholic beverages, including empirical studies, cost-effectiveness analyses, clinical guidelines, editorials, and historical correspondence. Given the scarcity of randomized controlled trials, the evidence base reflects a mixture of expert opinion, historical policy debates, and observational findings.

Of the literature reviewed, a subset of 25 publications explicitly discussed the possibility of fortifying alcoholic beverages with thiamine. These include both supportive and critical perspectives, ranging from early proposals in the 1940s to policy debates in Australia in the 1980s and beyond. The following sections synthesize the historical development of this debate, highlight the key arguments raised, and trace the reasons for its eventual marginalization. An overview of these publications and their respective positions is provided in Table 1.

**Table 1:** Examined articles and their position of alcoholic beverage fortification.

Article	Year	Type	Findings	Stance on fortification of alcoholic beverages	Counter arguments used for dismissal
Myerson et al.	1942	Review	Suggestion of education campaigns on the connection of WE and AUD, oral supplementation and beverage fortification.	Supportive	
Novak and Adams	1943	Experimental Study	Thiamine and nicotinic acid were found to be stable in whiskey.	Supportive	
Centerwall and Criqui	1978	Cost-benefit analysis	In the US, beverage fortification was considered economically advantageous with a cost-benefit ratio of 1:23–1:4.	Supportive	
Price and Theodoros	1979	Review	No issue in regards to practicability of fortification was found.	Supportive	
Weller	1984	Correspondence	Suggestion of “compulsory thiamine addition” to alcoholic beverages.	Supportive	
Price	1985	Invited Review	Suggestion to implement either a trial period of beverage fortification or publicity measures to highlight the necessity of thiamine supplementation.	Supportive	
Hambidge	1985	Correspondence	The effectiveness of oral supplementation in AUD was questioned.	Dismissive	Question of resorption. Fear of increased alcohol consumption.
Yellowlees	1986	Personal Viewpoint	Call for the fortification of both flour and alcoholic beverages followed by the proposal of a study 5 years after implementation.	Supportive	
Price et al.	1987	Review	Fortification of flour alone was considered as insufficient based on diet data in AUD. Suggestion of a trial period of beverage fortification followed by reevaluation.	Supportive	
van der Westhuyzen et al.	1987	Cross-sectional Study	Thiamine deficiency was found in a socially isolated group of workers in South Africa with increased alcohol intake. Fortification of a local beer was suggested.	Supportive	
Fischer and Yellowlees	1989	Cost-benefit Analysis	Cost-benefit ratio in favor of fortification in Australia.	Supportive	
Harper et al.	1989	Short Report	Necropsy data points to an increased rate of WKS in Australia with many cases being overlooked.	Supportive	
Binns et al.	1989	Review	Nutritionists perspective on the Australian debate on fortification suggesting caution.	Dismissive	Fear of increased alcohol consumption. Uncertainty of connection between thiamine, WE and KS.
Wodak et al.	1990	Conference Report	A consensus among physicians and nutritionists suggested a more cautious approach to fortification measures. They agreed on fortifying flour and cereals instead of alcoholic beverages.	Neutral to supportive	Mass medication. Harm to export goods. Uncertainty of connection between thiamine, WE and KS. Fear of increased alcohol consumption.
Connelly and Price	1996	Cost-effectiveness Analysis	The cost of averted cases of WE was estimated to be 28 times higher through the implemented approach of flour fortification compared to the fortification of beverages.	Supportive	
Drew and Truswell	1998	Editorial	Call for the reconsideration of beverage fortification.	Supportive	
Harper et al.	1998	Prospective Autopsy Study	Positive effect of flour fortification, suggestion of a cautious approach towards further fortification measures.	Neutral	
Bovet et al.	1998	Cross-sectional Population Study	Reduced blood levels of thiamine were found in subjects from the Seychelles. Fortification was suggested. It is falsely claimed that beverage fortification has been implemented in other countries.	Supportive	
Thomson	2000	Review	Review of the mechanisms by which thiamine is reduced in AUD and the connection to WKS.	Dismissive	Question of resorption. Problem of other vitamin deficiencies.
Harper	2006	Review	WKS was found to be underdiagnosed and undertreated. Preventative approaches are highlighted.	Supportive	
Kamien	2006	Viewpoint	Historical perspective on repeating patterns of objections to fortification measures.	Supportive	
Thomson and Marshall	2006	Non-systematic Literature Review	Patients with AUD should be treated with parenteral thiamine.	Dismissive	Question of resorption.
Galvin et al.	2010	EFNS Guidelines	Recommendations regarding diagnostic approaches of WE and treatment. Focus on parenteral options.	Dismissive	Question of resorption.
Whitfield et al.	2021	Review	Comparison of different targets of thiamine fortification. Passing mention that the fortification of alcoholic beverages is not permitted.	Dismissive	Question of legality.

## 2 Discussion focused on Australia

Discussions about the fortification of alcoholic beverages with thiamine began in the United States, emerging sporadically not long after the connection between thiamine deficiency and Wernicke–Korsakoff syndrome had become more widely understood. While early contributions—particularly in the 1940s and again in the late 1970s—focused on biochemical feasibility and cost-benefit considerations, the idea was never pursued in policy terms. In contrast, the topic gained considerably more momentum in Australia, where the high prevalence of WE in certain regions turned the question of fortification into a subject of active public health debate.

In 1978, a detailed economic argument for fortifying alcoholic beverages was published in the New England Journal of Medicine: Centerwall and Criqui calculated the cost of fortifying alcoholic beverages in the US, estimating a cost-benefit ratio ranging from 1:23–1:4. Although already striking, these figures were described by the authors as “conservative,” since they excluded a range of additional benefits from thiamine supplementation. Regarding possible objections, the authors pointed to thiamine naturally occurring in foodstuffs and it being already added to food like bread and milk in the US. Furthermore, they suggested that whether a beverage was fortified with thiamine would not influence rates of AUD (Centerwall and Criqui, 1978). According to the authors, the first call for the fortification of alcoholic beverages in the US stemmed from Novak and Adams, dating back to 1943 (Novak and Adams, 1943), who focused on determining whether vitamins would remain stable if added to alcoholic beverages. Following their citation trail, the original idea to fortify drinks seems to originate from Myerson et al., dating back to 1942. They also suggested campaigns to inform people with AUD of the need to supplement certain vitamins and proposed that the retailers of alcoholic beverages could provide said vitamins, as well (Myerson et al., 1942).

It was in Australia, however, that the idea received more systematic attention and practical exploration. Thiamine fortification was a significant topic of discussion in Australia starting in the 1970s, driven by the high prevalence of WE in the country, particularly in the Queensland region, due to various socio-economic and nutritional factors (Price, 1985). A 1979 article by Price and Theodoros discussed the challenges of fortifying alcoholic beverages with thiamine. The authors suggested calculating cost-benefit ratio, the stability of thiamine, and palatability for each type of beverage separately in regards to the paper by Centerwall and Criqui (Price and Theodoros, 1979).

In his 1985 review, Price summarized a series of earlier studies on the feasibility of fortifying beer with thiamine. He reported that no relevant changes in taste were observed in trials conducted in 1979 and 1981. Experiments also showed that allithiamines—initially considered as candidates due to their higher bioavailability—were unsuitable, as their sulfur bonds were broken down in beer, converting them into regular thiamine. Finally, Price noted that public surveys carried out after wide media coverage revealed little opposition to the idea, and that individuals who consume beer themselves were generally supportive of the proposed fortification (Price, 1985).

The idea of supplementing alcoholic beverages with thiamine was not limited to Australia. In 1984, Weller had argued for the supplementation of alcoholic beverages with thiamine, likening it to the addition of vitamins A and D to margarine (Weller, 1984). This led to D. M. Hambidge voicing concern in the same correspondence section of the the British Journal of Psychiatry that the supplementation would be seen by people with AUD as making drinking safer and more appropriate (Hambidge, 1985).

In 1986, Yellowlees continued the call for the fortification of bread flour and alcoholic beverages and summarized the then-current state of affairs with a focus on Australia in his literature review (Yellowlees, 1986). He attributed the start of the fortification debate to a speech held by Australian psychiatrist E. C. Dax at the first Leonard Ball Oration in 1968. Yellowlees reported that the Australian Associated Brewers expressed readiness to accept measures of fortification but would not initiate them. He further pointed out that vitamin C was already added to beer to “stabilize the frothy head.” Beyond that, he referred to a cost-benefit analysis which was not finished at the time of his writing his review, which appeared likely to show a “considerably greater” figure than Centerwall’s. He recommended (i) political action to fortify alcoholic beverages, flour, and bread with thiamine, (ii) the cooperation of nutritionists, physicians, and the alcohol industry to determine the needed thiamine levels, and (iii) the funding of a study that evaluates the results 5 years after the implementation of fortification measures.

In 1987, Price et al. outlined three strategies to address thiamine deficiency among people with AUD: fortifying flour, fortifying alcoholic beverages, and restoring thiamine in such beverages to offset carbohydrate metabolism. They saw the greatest preventive potential in fortifying beer (and possibly cheap wine), suggesting a dose of 0.5 mg per 740 mL bottle.

In the same article, they hypothesized that the “wide [media] publicity” surrounding fortification may have already led to increased thiamine supplement use among those at risk of thiamine deficiency due to AUD, pointing to a decline in WKS admissions to psychiatric hospitals as possible evidence (Price et al., 1987). Still, a necropsy study by Harper et al. reported a WKS prevalence of 2.1 % among Sydney adults and called for fortifying not only flour but also alcoholic beverages as a more direct and targeted strategy (Harper et al., 1989).

The question of thiamine fortification continued to surface in international contexts as well. A 1987 South African article described thiamine deficiency in an at-risk subpopulation with socio-economic similarities to those described by Price and colleagues. The fortification of a regional beer variety was suggested (van der Westhuyzen et al., 1987).

Critical perspectives on beverage fortification also began to emerge. In their 1989 article, Binns et al. discussed the risks associated with fortifying alcoholic beverages. They considered thiamine a "less certain" cause of KS and therefore disregarded it in their considerations. Based on their claim of uncertainty between WE and KS and their fears of the consequences of fortification on alcohol consumption, they suggested the fortification of just flour and the pursuit of health-promotion activities (Binns et al., 1989).

Soon after, Fischer and Yellowlees estimated the cost-benefit ratio of beer fortification as ranging from 1:30–1:6. They modified the earlier calculations of Centerwall and Criqui and applied it to the Australian figures of prevalence and incidence—arriving at estimates of a similar magnitude. Costs generated by thiamine deficiency, WE, and KS beyond long-term institutionalization (e.g., loss of earning power, short-term institutionalization) were not considered based on the assumption that patients suffering from AUD would generate these with or without thiamine deficiency. They based their calculations on Price’s estimate that about 20 % of WE cases could be prevented through the fortification of beer and bulk wine (Fischer and Yellowlees, 1989).

The Australian debate ultimately culminated in a national workshop convened in 1988 to weigh the arguments for and against thiamine enrichment (Wodak et al., 1990). Physicians and nutritionists engaged in the discussion. Both sides considered the ability of patients with chronic and excessive alcohol consumption to absorb thiamine orally to be “satisfactory,” while admitting that malabsorption might be a “significant problem” for a “small proportion” of the at-risk population. While it was accepted that the monetary benefits would exceed the costs, it was argued that enriched products might encounter difficulties when exported to other countries without similar policies. While a consensus could not be reached in regards to the fortification of alcoholic beverages—with medical disciplines being in support and the group of nutritionists and dietitians arguing against—strong support for the fortification of cereals (including flour) was found (Wodak et al., 1990). Beginning January 1, 1991 bread-making flour in Australia was fortified with thiamine, based on the recommendations of the workshop (Connelly and Price, 1996).

## 3 Development after the decision against the fortification of beer in Australia

After the decision to fortify bread-making flour instead of alcoholic beverages in Australia in 1991, the debate on targeted thiamine fortification continued in both scientific and public health discussions. This section traces how the topic evolved in the subsequent years—from cost-effectiveness analyses and emerging empirical data to shifting priorities in treatment strategies and guideline recommendations. By examining publications from the mid-1990s onward, we aim to highlight how arguments changed, which objections persisted, and why the proposal ultimately lost momentum despite new supporting evidence.

One of the earliest post-decision evaluations focused on the cost-effectiveness of different fortification strategies. Connelly and Price criticized the 1991 fortification of bread-making flour for its poor cost-effectiveness. Based on consumption data from 40 individuals with AUD—specifically their average intake of bread and full-strength beer—they modelled three scenarios: (i) flour fortification, (ii) beer fortification, and (iii) beer plus lower-priced wine. Assuming 500 annual WE cases, they estimated that flour fortification was at least 28 times more expensive per averted case than fortifying beer alone (Connelly and Price, 1996).

In parallel, clinical and editorial voices also began to reflect on the observed effects of the implemented policy. A 1998 editorial in the Medical Journal of Australia reported a correlation between the implemented fortification of bread flour and a reduction in Australian WE cases. The authors argued that WE cases persist and could be better-addressed by the fortification of beer (Drew and Truswell, 1998). Harper et al., in a 1998 study of 2212 autopsies conducted six years after the introduction of flour fortification, reported a significant decline in WKS prevalence compared to earlier data. They discussed several potential explanations for this decrease, including improved thiamine intake due to fortification, general health improvements, and increased clinical awareness among healthcare providers. At the same time, they emphasized that six years of follow-up were insufficient to draw firm conclusions, and proposed a longer-term reassessment to determine whether the observed trend would persist and whether additional measures, such as the fortification of alcoholic beverages, might be warranted (Harper et al., 1998).

Around the turn of the millennium, the question of thiamine absorption during ongoing alcohol use increasingly became the dominant objection raised in discussions surrounding fortification.A German article from the year 2000 by Hinze-Selch et al. cited the mentioned possibility of reduced incidence of WE following flour fortification in Australia and suggested the oral supplementation of people with AUD and other at-risk patients. The option of implementing similar measures in Germany was not mentioned, nor was the fortification of alcoholic beverages (Hinze-Selch et al., 2000). The article cited a 1970 study by Thomson et al. (Thomson et al., 1970) to assert that thiamine resorption ceases completely when alcohol consumption is continued. However, the cited study does not support such a categorical interpretation. In that study, nine participants who received oral ethanol and radiolabeled thiamine simultaneously exhibited a 70 % reduction in expected serum radioactivity. This value reflects an indirect measure of thiamine uptake and indicates a substantial—though not complete—impairment of absorption under the influence of alcohol (Thomson et al., 1970). In his original call for fortification, Price cited the same study to estimate that at least six times the normal amount of thiamine would be needed to overcome this degree of malabsorption (Price and Theodoros, 1979).

In Australia, the debate on beverage fortification continued with both supportive and skeptical voices. In his 2000 article, Allan D. Thomson pointed to a possible decline in WE cases following flour fortification in Australia and acknowledged the potential of beer fortification. At the same time, he questioned its merit by arguing that such a measure would not address other nutritional deficiencies common in AUD—such as pyridoxine, niacin, proteins, and minerals. However, this line of reasoning risks dismissing a targeted intervention on the grounds that it does not solve broader nutritional issues. As such, it appears less as a scientific counterargument than as a shift of focus away from the specific problem of thiamine deficiency (Thomson, 2000).

In his 2006 review publication, C. Harper suggested that fortification procedures as implemented in Australia should also be considered in other countries. He used the term “disappointing” for the fact that the choice fell against the fortification of beer (Harper, 2006). He pointed to additional benefits (e.g., improvement of working memory) of thiamine supplementation in AUD patients as described by Ambrose et al. beyond the prevention of WE (Ambrose et al., 2001). In the same year, Australian physician Max Kamien compared the objections made in regard to the fortification of alcoholic beverages to those made whenever other issues of supplementation—which had long been successfully implemented—were first discussed. He pointed out that objections were often based on “self-interest and tunnel vision,” with each side employing the medical principle which best supported their position. He suggested that rhetoric should be avoided in favor of research under consideration of previous objections in similar discussions (Kamien, 2006).

Another non-systematic review from Thomson and Marshall emphasized the superiority of IV or IM thiamine over oral options in at-risk patients. The authors highlighted the need for further studies into the fortification of beverages before such measures could be “adopted or discarded.” Among their concerns were the unresolved question of sufficient absorption and the risk of sending a misleading message to AUD patients and physicians, potentially leading to a “false sense of security.” At the same time, they acknowledged that around 90 % of WE cases go undiagnosed—highlighting a significant gap in early detection and intervention (Thomson and Marshall, 2006).

From 2010 onward, mentions of beverage fortification have become markedly rarer in the literature, and where present, tend to reflect a more categorical rejection of the approach.

The 2010 EFNS guidelines for diagnosis, treatment, and prevention of WE addressed the fortification of alcoholic beverages and dismissed it as “useless.” The authors mentioned the reduced absorption and made the claim that “during alcohol metabolism, thiamine will neither be phosphorylated nor incorporated to enzymes in body tissues (Galvin et al., 2010).” They cited a 1980 article by Hoyumpa which lists a variety of mechanisms leading to malabsorption of thiamine both in the presence of ethanol and in organisms accustomed to continued alcohol consumption. However, the cited article is a mechanistic review outlining several pathways by which alcohol may impair thiamine uptake, particularly via inhibition of active transport. It does not claim that absorption is entirely blocked; rather, it notes that passive absorption may still occur, especially at higher concentrations (Hoyumpa, 1980). Nonetheless, the option of fortification was dismissed as “useless” in the EFNS guidelines (Galvin et al., 2010).

Only one article published after 2010 in which the fortification of beer or other alcoholic beverages gets mentioned was found. A 2021 narrative review by Whitfield et al. briefly mentioned the past discussion on the fortification of beer and listed the fact that fortification of alcoholic beverages is forbidden by the Australia New Zealand Food Standards Code as the reason why it was not implemented (Whitfield et al., 2021).

## 4 Discussion

### 4.1 Assessing the counterarguments

In the following sections, the arguments against the fortification of alcoholic beverages with thiamine are examined. These arguments are reassessed in the context of the entire debate and contemporary scientific understanding to gauge their validity for a possible revival of the topic.

#### 4.1.1 Issue of resorption

A recurring point of contention has been the resorption of thiamine alongside continued alcohol consumption. Several authors (Cook et al., 1998, Hambidge, 1985, Thomson, 2000, Thomson and Marshall, 2006) dismissed the idea of fortifying beer or other alcoholic beverages, arguing that alcohol impairs thiamine absorption too severely. The 2010 EFNS guidelines for WE expand on the argument by pointing to the reduction of not just absorption but also phosphorylation and incorporation of thiamine into body enzymes (Galvin et al., 2010). However, the suggestion to counteract these effects through sufficiently high concentrations of thiamine in the fortified medium had already been proposed earlier (Yellowlees, 1986). Across all these arguments, the well-established impairment of absorption and utilization is often treated as if it implied a complete failure of thiamine uptake. Yet this assumption appears difficult to reconcile with the clinical reality: most individuals with AUD do not develop WKS. Notably, two authors responsible for several of the critical articles disclosed a monetary connection to the producer of a widely used parenteral thiamine supplement (Thomson and Marshall, 2006). The sources cited to substantiate this claim of ineffectiveness preceded the discussion about fortification and can be assumed to have been considered by the lead proponents of the fortification measures. Already in 2006, Max Kamien warned that opposition to fortification might stem from self-interest or tunnel vision—a concern that remains relevant in light of the above (Kamien, 2006).

#### 4.1.2 Question of legality

The earliest-found suggestion of beverage fortification points to the necessity of legislative changes judging this obstacle to be easily overcome (Myerson et al., 1942). While existing regulations on food fortification may present a valid obstacle, they should not be treated as a reason to curtail further research or to dismiss the option altogether, as appears to be the case in the most recent publication mentioning the topic (Whitfield et al., 2021).

#### 4.1.3 Problem of other vitamin and nutrient deficiencies not being addressed

As noted earlier, concerns have been raised that thiamine fortification alone would not address other nutritional deficiencies in individuals with AUD (Thomson, 2000). While this does not undermine its targeted purpose, it draws attention to broader gaps—such as the often-overlooked deficiency in vitamin C, which may persist even when routine nutrients and vitamins are supplemented (Marik and Liggett, 2019).

#### 4.1.4 Promotion of consumption

The earliest suggestion of beverage fortification by Myerson et al. expected the counterargument that the supplementation of alcoholic beverages might be seen as an encouragement of AUD. The authors dismissed this scenario as unlikely and hypothesized a reduction of alcohol consumption through the awareness accompanying supplementation in the long run (Myerson et al., 1942). Similarly, Centerwall and Criqui mentioned the possibility of fortification leading to alcohol gaining an “undeserved aura of legitimacy” as a food, which they dismissed as not “cogent” and pointed to it being the main source of calories for “millions of Americans.” They also expected the argument that alcohol consumption might increase due to it being considered “safer”, which they considered unlikely (Centerwall and Criqui, 1978).

Another iteration of this argument was made by Hambidge in 1985 and later again by Thomson and Marshall in 2006: The supplementation of thiamine through the consumption of beer or other beverages could create a “false sense of security” in both patients and physicians, as put by Thomson and Marshall (Thomson and Marshall, 2006). Patients might assume that all the problems arising from alcohol consumption might be prevented by the fortification measure, so feared Hambidge (1985), as well as Binns et al. (1989). The latter projected a “disaster” arising from increased alcohol consumption and its related diseases not caused by thiamine deficiency. They further expressed their fears that the alcohol industry would aggravate this with advertisements focused on the health benefits of alcohol consumption. This argument—that the fortification of alcohol would result in increased consumption—was repeated at the crucial 1989 workshop, as reported by Wodak et al. (1990). To the knowledge of the authors, there are no known cases of alcoholic beverages being voluntarily enriched with thiamine, whether motivated by concern for consumers' health or to bolster sales through the controversial marketing of their product as "healthy." Admittedly, this could be attributed to the strict regulations governing additives in alcoholic beverages. Nonetheless, widespread fortification would likely require changes to foodstuff regulations. The legislation could include a provision that fortification may not be used for advertising purposes.

#### 4.1.5 Feared damage to alcoholic beverage exports

A change of regulations regarding the fortification of alcoholic beverages might lead to problems when exporting said beverages (Kamien, 2006, Wodak et al., 1990). During the workshop where this argument was brought up, it was countered that there could be different procedures and standards for exported and domestic goods (Wodak et al., 1990).

#### 4.1.6 Mass medication

Among the dietitians and nutritionists at the 1988 workshop, concern was voiced that the considered approach of fortification would be “mass medication,” with many people being given a substance to treat only a few (Wodak et al., 1990). Cost-effectiveness was questioned in this regard, despite prior and subsequent economic evaluations estimating a substantial benefit-to-cost ratio in favor of beverage fortification (Centerwall and Criqui, 1978, Connelly and Price, 1996, Fischer and Yellowlees, 1989).

#### 4.1.7 Uncertainties regarding the connection between WE and KS

One of the counterarguments Yellowlees mentioned in his article (Yellowlees, 1986) was that posed by Wood et al. (Wood et al., 1986), whom he considered to have a more “conservative view” on the topic: They pointed to the lack of proof that KS resulted from thiamine deficiency while admitting the “close clinical relation to WE.” The studies that would be required to provide definitive proof were considered unethical to conduct in humans, and results from animal models—such as rhesus monkeys—were deemed unreliable due to significant neuroanatomical differences, particularly in the structure of the third ventricle (Yellowlees, 1986). Uncertainty about the relationship between WE and KS was also claimed by Australian nutritionists Binns et al., who further pointed to the body’s thiamine stores lasting up to 5 weeks and thus longer than “most alcoholic binges” (Binns et al., 1989). Here, the authors failed to grasp the reality of the lifestyle of many patients suffering from AUD, who often meet their entire caloric requirements through alcohol consumption for extended periods of time.

The nutritionists repeated the doubt about the connection between WE and KS in the discussion at the 1988 workshop which culminated in the compromise to fortify bread and not beer (Wodak et al., 1990). A subsequent review by M. D. Kopelman pointed to the fact that Korsakoff’s own case series included 15 cases of non-alcoholic origin; the author considered thiamine depletion to be “almost certainly” the cause of both WE and WKS (Kopelman, 1995).

### 4.2 Limitations

It remains uncertain whether the fortification of alcoholic beverages might inadvertently encourage increased alcohol consumption due to the perception of reduced risks of alcohol intake. The effectiveness of these measures in preventing cases of WKS and their actual impact on mortality and morbidity remain unclear. A limitation of the current study lies in its reliance on narrative sources, which lack robust empirical validation. The absence of pilot studies has left only estimations regarding the technical and legislative feasibility, as well as the measurable outcomes, of fortification. Furthermore, no recent cost-benefit analyses were available to evaluate the economic viability and overall efficacy of possible fortification measures. In addition, there were limited data regarding the appropriate dosage, benefits, and application of thiamine for the prophylaxis of WKS, which further complicate the assessment of the potential effectiveness of such interventions.

## 5 Conclusion

Drawing from the reconstruction of events, it becomes evident that, in the case of Australia, the proposal to fortify alcoholic beverages was closely considered but ultimately declined in favor of a more conservative approach. This decision was primarily founded on ethical concerns and fears of adverse effects. Since then, the option appears to get dismissed or ignored altogether because of claimed or perceived ineffectiveness. This perspective often originated from an ongoing debate over the superiority of intramuscular injections compared to oral supplementation options, which has further complicated the discussion.

Even though oral supplementation might be inferior to parenteral injection, the issue of application becomes moot if patients do not reach hospitals or clinics. Completely dismissing oral supplementation for AUD patients who continue to consume alcohol,as done by many authors (Cook et al., 1998, Hinze-Selch et al., 2000), is dangerous and harmful if the alternative is that the group of patients most at risk of developing WE receives no thiamine at all. Fortification should not be considered as an alternative to established supplementation in clinically apparent cases of thiamine deficiency but as an additional preventative measure.

Historically, vitamin deficiencies were best-treated when they were prevented from occurring (Lanska, 2010). A possible reason why fortification has been overlooked might be that its implementation and effects are so commonplace that we no longer consider it an option. The primary challenge associated with potential fortification lies in the magnitude of the undertaking. Nationwide efforts have historically required substantial momentum—as seen in the 1940s, when the US fortified flour and bread with thiamin, niacin, riboflavin, and iron, primarily to improve young men's nutrition and increase the number of military recruits fit for service (Lanska, 2010).

Critics are likely to remain unconvinced until definitive data are available, which could probably only be collected retrospectively. Given the lack of evidence of thiamine’s toxicity even at exceedingly high doses and the successes of other historic fortification strategies, the approach of implementing it as a trial appears justifiable. It is particularly unfortunate that the ideal situation—a motivated, isolated country ready to implement comprehensive measures in this regard—narrowly missed the opportunity to explore this option.

## CRediT authorship contribution statement

**Georg Juckel:** Writing – review & editing, Supervision, Methodology, Conceptualization. **Thomas Jedamzik:** Writing – original draft, Formal analysis, Data curation, Conceptualization.

## Contributors

TJ and GJ designed the study. TJ conducted the literature search and analysis. TJ wrote the first draft. GJ revised the manuscript. All authors contributed to and approved the final manuscript.

## Funding

This research was not funded by any specific grant from funding agencies in the public, commercial, or non-profit sectors.

## Declaration of Competing Interest

The authors declare that they have no known competing financial interests or personal relationships that could have appeared to influence the work reported in this paper.
